# Promising Therapeutic Potential of miR-220-3p Mimic Against Murine Trimethyltin Hippocampal Injury

**DOI:** 10.5812/ijpr-165755

**Published:** 2026-04-25

**Authors:** Roya Khosh Ravesh, Vahid Khodashenas, Mina Goudarzi, Leila Mohammadi, Tourandokht Baluchnejadmojarad, Mehrdad Roghani

**Affiliations:** 1 Iran University of Medical Sciences, Tehran, Iran; 2 Neurophysiology Research Center, Shahed University, Tehran, Iran

**Keywords:** Alzheimer’s Disease, Trimethyltin, miR-220-3p, Oxidative Stress, Neuroinflammation, Pyroptosis, Apoptosis

## Abstract

**Background:**

Trimethyltin (TMT) is an organotin compound known to induce neurotoxicity within the limbic system of the brain, particularly in the hippocampal region, with neurodegenerative changes resembling those of Alzheimer’s disease (AD).

**Objectives:**

This investigation examined the impact of miRNA-220-3p (miR-220-3p) on TMT-induced neurotoxicity and associated behavioral abnormalities, such as spatial learning and memory impairments, and identified the potential molecular mechanisms.

**Methods:**

To induce neurotoxicity, TMT was injected (8 mg/kg, i.p. once), and after 1 hour, miR-220-3p was microinjected intraventricularly (ICV route, once) for the possible mitigation of TMT-induced neurotoxicity. Different behavioral assessments were employed to determine spatial learning and memory function. Moreover, hippocampal measurements of brain-derived neurotrophic factor (BDNF) and Sirtuin-1, oxidative stress-, apoptosis-, and neuroinflammation-related factors, and histochemical changes were performed.

**Results:**

The TMT injection led to behavioral abnormalities in the novel object discrimination and Barnes maze tests, heightened oxidative stress [elevating reactive oxygen species (ROS) levels, nitrite, and lipid peroxidation and decreasing the activities of antioxidant enzymes, including catalase (CAT) and superoxide dismutase (SOD)], reduced BDNF and Sirtuin-1 levels, increased neuroinflammation [escalating the secretion of pro-inflammatory mediators, including tumor necrosis factor-α (TNF-α) and interleukin-6 (IL-6)], raised activities of acetylcholinesterase (AChE), myeloperoxidase (MPO), beta-secretase 1 (BACE-1), caspase-1, and caspase-3, accompanied by a reduced number of CA1 pyramidal neurons and higher glial fibrillary acidic protein (GFAP) immunoreactivity. In contrast, microinjection of miR-220-3p reversed most of these alterations.

**Conclusions:**

The findings of this investigation imply that miR-220-3p may mitigate TMT-induced neurotoxicity, which is attributed to the suppression of hippocampal oxidative stress, neuroinflammation, and caspase-dependent apoptosis and pyroptosis, and part of its beneficial effect is associated with the upregulation of BDNF and Sirtuin-1.

## 1. Background

Trimethyltin (TMT) is an organotin compound and a neurotoxin that is usually employed as a stabilizer and biocide in industry and agriculture (1). Owing to its potential role in the initiation of toxicity in humans (especially in the hippocampal area of the brain) and ecosystems, TMT is a risk factor for safety in the food industry (2, 3). Tremor and convulsion can occur following TMT accumulation in cerebral tissue, which in turn is associated with derangements of excitability in the brain and behavioral abnormalities, including learning and memory dysfunction, hyperactivity, seizure-like behaviors, and aggression (4-7). Although some previous investigations have focused on exploring the possible mechanisms involved in TMT-induced neurotoxicity, the precise etiological causes and promising therapeutic approaches have not yet been fully elucidated. The TMT is associated with the elevation of intracellular Ca^2+^ levels through excitotoxicity in neuronal cells, disturbances in Ca^2+^ homeostasis, mitochondrial and endoplasmic reticulum dysfunction, membrane destruction, cytoskeletal alterations, and apoptotic cell death owing to the excessive generation of reactive oxygen species (ROS) and reactive nitrogen species (RNS), followed by the release of pro-inflammatory cytokines (PCs) (7-11). According to existing literature, the hippocampal CA1, CA3, and dentate areas are the main targets of TMT in rats (12).

Although numerous studies have employed natural compounds to achieve neuroprotection (13-15), there remains a need to explore novel therapeutic agents with greater efficacy. MicroRNAs (miRNAs or miRs) are short, non-coding RNA molecules that play pivotal roles in post-transcriptional gene expression. Altered expression of miRNAs has been implicated in the etiology of various neurological disorders, such as Alzheimer’s disease (AD). miRNAs are central to controlling apoptosis and pyroptosis, as well as oxidative and inflammatory alterations, and accordingly may exert neuroprotective effects (16, 17). In addition, miRNAs can be regarded as biomarkers of various neurological disorders (16). In recent years, miRNAs and their mimics have been postulated as potential innovative strategies for the therapeutic management of incorrigible CNS diseases (18, 19). miRNA-220 is one of the key miRNAs that is released under hypoxic conditions and displays a potent role in the synthesis of capillary-like structures from venous endothelial cells (20). However, there is no report on its potential beneficial properties.

## 2. Objectives

This study was designed to examine the potential therapeutic impact of miRNA-220-3p (miR-220-3p) on TMT-induced neurotoxicity and behavioral abnormalities in male Wistar rats.

## 3. Methods

### 3.1. Animals

Forty male Wistar rats (12 weeks of age), with an average weight of 195 - 230 g, were obtained from the Animal Laboratory Center of Iran University (Tehran, Iran). The animals were kept under controlled conditions of temperature (21 - 23°C) and humidity (40 - 47%), with a 12 h light cycle and free access to standard food and tap water. All experimental protocols were approved by the Ethics Committee of Iran University of Medical Sciences in 2023 and were conducted in accordance with ARRIVE guidelines.

### 3.2. Experimental Protocol

The animals (n = 40) were randomly assigned into five groups: (1) Control, including healthy animals; (2) control + miR-220-3p, including healthy animals that received miR-220-3p mimic (with a sequence of UCCACCACAGUGUCAGAGACC, Accession no. MIMAT0012826 (miRBase), Cat no. B02003-rno-miR-220-3p, GenePharma, China) at a concentration of 2.5 µg/2.5 µL via intracerebroventricular injection; (3) TMT, including animals that received a single intraperitoneal injection of TMT-chloride (Cat no. sc-301942, Santa Cruz Biotech, USA), dissolved in normal saline at a concentration of 8 mg/kg (21); (4) TMT + miR-220-3p, including rats that received a single intraperitoneal injection of TMT-chloride dissolved in normal saline at a concentration of 8 mg/kg and, 1 hour later, were treated with miR-220-3p at a concentration of 2.5 µg/2.5 µL via intracerebroventricular injection; (5) TMT + NC mimic (MyBioSource, USA), including rats that received a single intraperitoneal injection of TMT-chloride dissolved in normal saline at a concentration of 8 mg/kg and, 1 hour later, were treated with a control miRNA with no known biological activity.

To assess hippocampal changes in miR-220-3p levels after its microinjection, we conducted a separate experiment on 10 rats in two subgroups, i.e., control and miR-220-3p-treated control, and after one day, its hippocampal expression was determined.

For stereotaxic surgery, rats were anesthetized with ketamine/xylazine (90 mg/kg and 10 mg/kg, respectively), fixed in a Stoelting stereotaxic device, the scalp was shaved, and a hole was drilled at AP: -0.8 mm, ML: 1.3 mm, and DV: 3.7 mm, all relative to the Bregma point. Thereafter, miR-220-3p was dissolved in RNase-free artificial CSF and injected at 2.5 µg/2.5 µL at a flow rate of 1 µL/min using a 5 µL Hamilton syringe.

### 3.3. Novel Object Discrimination

NOD was performed in the third week and consisted of two sessions, with an interval of 4 h. In the first session, the animals were exposed to two identical objects for 5 min. After 4 h, one of the objects was randomly replaced with a novel object, and the exploration of each object was monitored in both sessions. The time spent exploring the novel object in comparison with the total time was considered the Discrimination Index (21). The test was performed by an examiner who was unaware of the experimental design.

### 3.4. Barnes Maze

The Barnes maze was employed to assess spatial memory. This maze comprises a flat circular field (120 cm in diameter) positioned 100 cm above the floor. It possesses twenty holes (10 cm in diameter) around its periphery. To provide an aversive stimulus, a light bulb was fixed in the middle. This test consists of two sessions, including training and probe. In each session, the animal was allowed to explore for 2 min. After training for escape from the lit arena and learning the position of the escape box, the probe test was performed, and the latency to explore the escape box and the number of errors (including exploration of holes other than the escape box) were calculated as described in a previous investigation (21). An examiner unaware of the experimental design performed the test.

### 3.5. Sample Preparation

After sacrificing the animals at the end of the behavioral assessments under CO_2_, the brain tissues were separated from the skull. The right hemispheres were used to prepare homogenates, and the left side was fixed in a 10% formalin solution for histochemical evaluations. After homogenizing the hippocampus in cold 150 mM Tris buffer (pH = 7.4), the samples were centrifuged at 5000 rpm for 15 min (4°C) to collect the supernatant for further assessment. Protein concentration in the samples was determined by the Bradford method, and normalization of biochemical findings was made accordingly, as has been mentioned before (22).

### 3.6. Hippocampal Oxidative Stress

A dichlorofluorescein diacetate probe with excitation at 488 nm and emission at 525 nm was employed to determine ROS levels. Inflammatory markers and oxidative stress factors were measured using ELISA kits, as described in the manufacturer’s instructions. A commercial kit from KiaZist, Iran (Cat # KMDA96) was utilized to assess malondialdehyde (MDA) levels. In brief, thiobarbituric acid was diluted in glacial acetic acid and heated at 95°C for 20 min, with tetraethoxypropane as the standard. The optical density of the pink MDA-TBA adduct was measured at 535 nm. The MDA concentration was expressed as nmol/mg of protein. Superoxide dismutase (SOD) activity was evaluated using a commercial kit (Cat # 706002, Cayman, USA). Briefly, a tetrazolium salt was utilized to identify superoxide radicals generated by xanthine oxidase and hypoxanthine. Thirty minutes after interaction between the sample, radical detector solution, and xanthine oxidase, and with erythrocyte SOD as the standard, the optical density was measured at 405 nm. The SOD activity was expressed as SOD activity/mg of protein. Catalase (CAT) activity was measured using a commercial kit (Cat # KCAT96, KiaZist, Iran) based on a prior investigation by our group (23). Findings were expressed as CAT activity/mg of protein. A commercial assay kit from Abcam, USA (Cat # ab273268) was employed to measure caspase-1 as a Pyroptosis Index. During the process, YVAD-p-NA served as the substrate, and the final product was the chromophore p-nitroanilide. The optical density was read at 405 nm, and the findings were expressed as ODs of the samples.

A commercial assay kit from Abcam, USA (Cat # ab39401) was utilized to determine caspase-3 as a marker of cell death. During measurement, DEVD-p-NA was employed as the substrate, and the final product was the chromophore p-nitroaniline. The optical density was read at 405 nm, and the findings were expressed as ODs of the samples. Griess reagent was utilized to measure nitrite levels as an indicator of nitric oxide amounts (24). Equal volumes of the sample and reagent, containing 1% sulfanilamide and 0.1% N-(1-naphthyl)ethylenediamine, were mixed in an acidic medium. After 15 min, the absorbance was read at 540 nm. Protein carbonyl was assessed based on a prior investigation (25). To this end, 10.1 mM DNPH was mixed with the samples, and the resulting solution was incubated at 25°C for one hour in a dark room. After adding a denaturing buffer containing heptane (99.5%), absolute ethanol, 150 mM sodium phosphate buffer (pH = 6.8), and 3% SDS, the specimens were vortexed and centrifuged at 6000 rpm for 15 min. To isolate protein, the samples were washed twice with ethyl acetate/ethanol at equal volumes. After dissolving the separated protein in denaturing buffer, the absorbance was read at 370 nm.

### 3.7. Acetylcholinesterase Activity

To measure acetylcholinesterase (AChE) activity, an AChE kit from Abcam, USA (Cat # ab138871) was utilized, with a reagent including acetylthiocholine and DTNB. The absorbance was read at 408 nm after 30 min. Findings were reported as nmol/min/mg of protein.

### 3.8. Inflammatory Markers, Brain-Derived Neurotrophic Factor and Sirtuin-1

Concentrations of tumor necrosis factor-α (TNF-α), interleukin-6 (IL-6), and IL-10, as well as Sirtuin-1, were determined by the sandwich ELISA method using specific primary and HRP-conjugated antibodies (obtained from Santa Cruz Biotech, USA). The BDNF levels were determined using a specific assay kit from MyBioSource (USA). Findings were expressed as pg/mg of tissue protein.

### 3.9. Assessment of Myeloperoxidase

The MPO activity was measured as an indicator of neutrophil infiltration. Assessment of MPO was performed using tetramethylbenzidine, 0.75 mM H_2_O_2_, and 0.18 M H_2_SO_4_ in several steps, and the absorbance was read at 450 nm (26).

### 3.10. Assessment of BACE 1 Activity

Beta-secretase 1 (BACE-1) activity was determined as described in a prior investigation (27). The reaction mixture included DL-BAPNA solution (6 mM) and sodium acetate buffer (50 mM, pH: 4.50). The reaction was performed for 65 min at 37°C, and the activity of BACE-1 was expressed as ΔA/h.

### 3.11. Nissl Staining

After processing and embedding in paraffin, tissue blocks were cut into coronal sections with a thickness of 5 μm using a rotary microtome. Several sections were stained with Cresyl violet acetate solution. Six fields at planes between -3.3 and -3.8 mm from the bregma were selected for cell counting. Cells with an obvious boundary and a visible nucleolus were counted. Quantitative analysis was performed using ImageJ 1.49 (NIH). A blinded examiner, unaware of the experimental design, performed the analysis.

### 3.12. Immunohistochemistry

After processing, embedding in paraffin, and preparing coronal sections with a thickness of 5 μm, some sections were subjected to a primary monoclonal antibody against glial fibrillary acidic protein (GFAP) (Cat # sc-166481; 1:60) and then exposed to a secondary HRP-conjugated antibody (1:70, Santa Cruz Biotech, USA). Afterwards, they were incubated with 3,3′-diaminobenzidine (Cat # sc-209686, Santa Cruz Biotech, USA) and hydrogen peroxide for visualization. Counterstaining of sections was performed using hematoxylin for 10 s. ImageJ 1.49 software (NIH) was used to determine GFAP immunoreactivity (IRA) in the stratum radiatum region at planes 3.3 - 3.8 mm posterior to the bregma. A blinded examiner performed the analysis.

### 3.13. RT-qPCR Assay

Total RNA was extracted using TRIzol solution (Kiazist, Iran), and the reverse transcription reaction was subsequently performed using the Takara Bio miRNA First Strand Synthesis Kit (Japan) according to the manufacturer’s instructions. Quantitative real-time polymerase chain reaction was conducted using TB Green Premix Ex Taq II (Takara, Japan) on an Applied Biosystems 7500 system (Thermo-Fisher Scientific, USA). Data analysis was performed using the 2^-ΔΔCt^ method. U6 was used as the control. The primers used for the RT-qPCR reaction were as follows: miR-220-3p, F: TCCACCACAGTGTCAGAGACC, R: GGTCTCTGACACTGTGGTGGA; U6, F: CTCGCTTCGGCAGCACAT, R: TTTGCGTGTCATCCTTGCG.

### 3.14. Statistical Analysis

GraphPad Prism 10.5.0 was utilized to carry out all statistical analyses. Results were presented as means ± SE. One-way ANOVA followed by Tukey’s test was employed to compare cohorts. An unpaired *t*-test was used for comparing data related to miR-220-3p gene expression. The Kolmogorov-Smirnov test was used to assess the normal distribution of quantitative variables. Outliers were detected using the Grubbs test. A P-value less than 0.05 was considered an indicator of significant differences.

## 4. Results

### 4.1. Behavioral Findings

**Figure 1.**
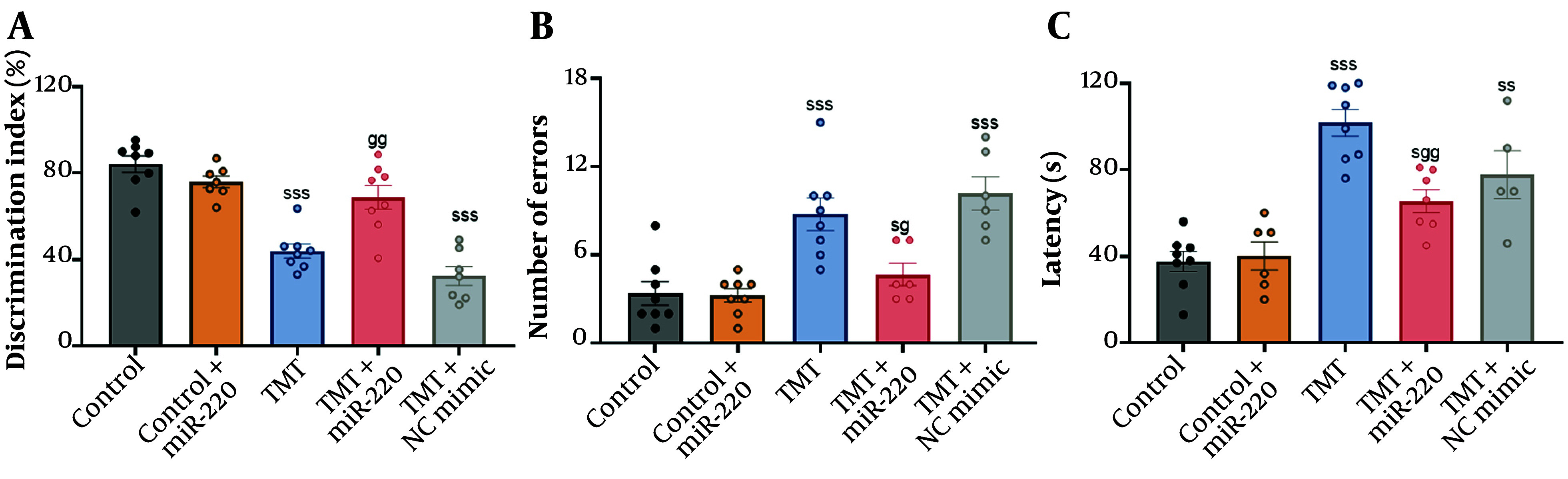
Impact of miRNA-220 against trimethyltin (TMT)-induced behavioral abnormalities: A, Discrimination Index in the NOD test, B, number of errors, and C, latency in the Barnes maze test (s P < 0.05, ss P < 0.01, and sss P < 0.001 vs. control; g P < 0.05, gg P < 0.01 vs. TMT).

Spatial short-term and recognition memory were measured by the NOD test. Our analysis showed significant inter-group differences using two-way ANOVA (Figure 1 a significant interaction of TMT versus miR-220-3p, F1, 34 = 22.89, P < 0.001; a significant miR-220-3p effect, P < 0.05; and a significant TMT effect, P < 0.001). Further analysis demonstrated that TMT-induced animals exhibited a significantly reduced Discrimination Index versus the control (P < 0.001, a decrease of 47.72%). Injection of miR-220-3p mimic significantly improved the Discrimination Index versus the TMT cohort (an increase of 56.20%, P < 0.01). Additionally, the TMT group microinjected with NC mimic did not show a significant improvement in this index compared with the TMT cohort (P > 0.05). Moreover, the miR-220-treated control did not show a significant change in the NOD Index, as compared to the control cohort (P > 0.05).

The Barnes maze test was used to assess hippocampus-dependent spatial and long-term cognitive functions. Our statistical assessment showed significant inter-group differences using two-way ANOVA (Figure 1 and C; for errors: A significant interaction of TMT versus miR-220-3p, F1, 32 = 7.68, P < 0.01; a significant miR-220-3p effect, P < 0.01; and a significant TMT effect, P < 0.001; for latency: A significant interaction of TMT versus miR-220-3p, F1, 30 = 5.14, P < 0.05; a non-significant miR-220-3p effect, P > 0.05; and a significant TMT effect, P < 0.001). Further assessment showed that the number of errors [F (4, 31) = 13.16, P < 0.001; an increase of 159.64%] and latency [F (4, 28) = 18.86, P < 0.001; an increase of 170.46%] were significantly higher in TMT-induced animals versus the control (P < 0.001). When compared with the TMT cohort, animals treated with miR-220-3p mimic demonstrated significant reductions in latency (a decrease of 35.70%; P < 0.01) and errors (a decrease of 46.74%; P < 0.05). Meanwhile, the TMT group microinjected with NC mimic did not show a significant reduction in errors and latency compared to the TMT cohort (P > 0.05). In addition, the miR-220-3p-treated control did not show a significant change in errors and latency, as compared to the control cohort (P > 0.05).

### 4.2. Oxidative Stress Factors

**Figure 2.**
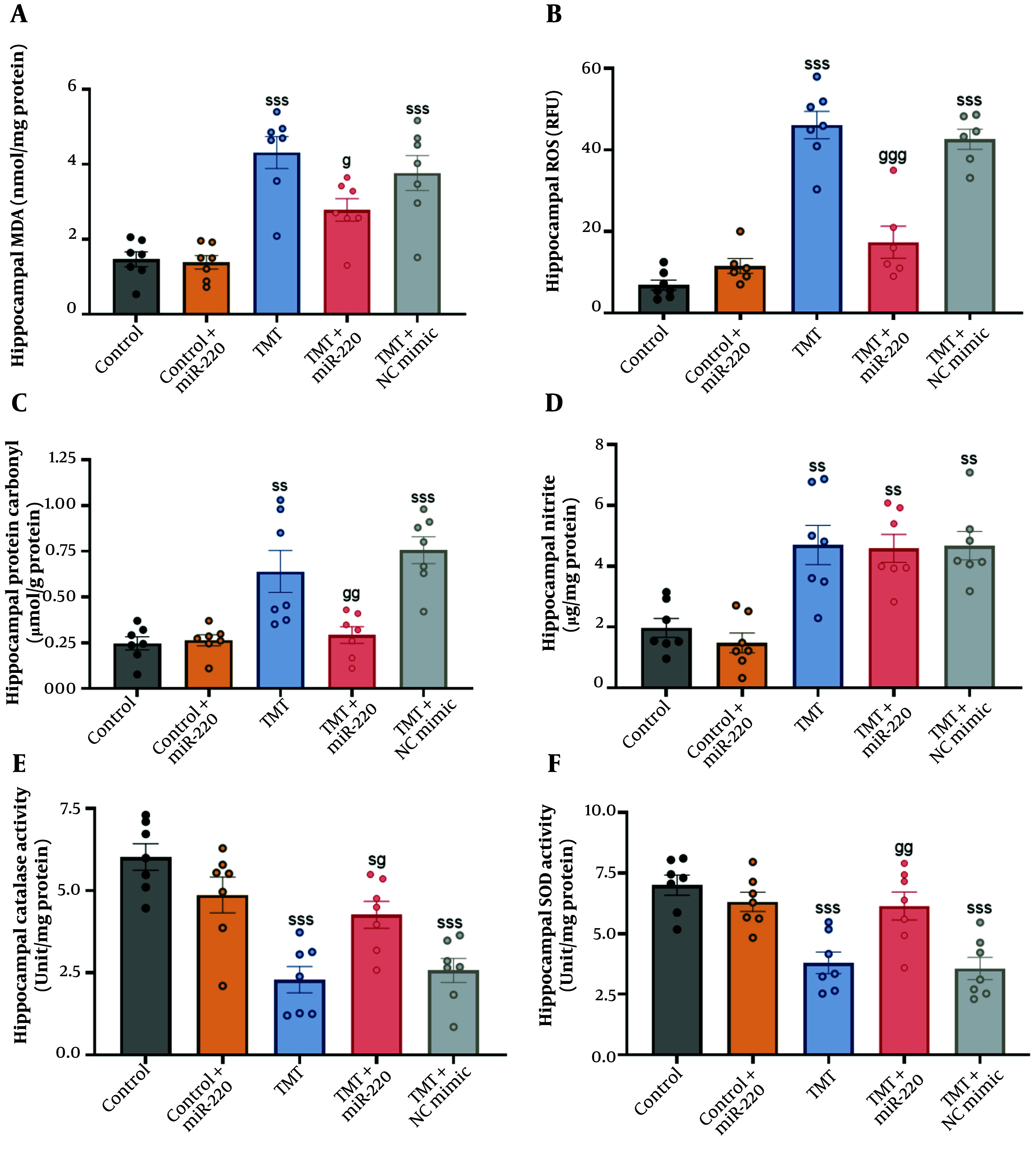
Impact of miRNA-220 on oxidative stress factors in the hippocampal region of trimethyltin (TMT)-induced animals: A, malondialdehyde (MDA); B, reactive oxygen species (ROS); C, protein carbonyl; D, nitrite; E, catalase (CAT); and F, superoxide dismutase (SOD) (s P < 0.05, ss P < 0.01, and sss P < 0.001 vs. control; g P < 0.05, gg P < 0.01, ggg P < 0.001 vs. TMT).

Hippocampal tissue was used to measure oxidative stress factors. Our statistical analysis showed significant inter-group differences using two-way ANOVA (Figure 2A-F; for MDA: A non-significant interaction of TMT versus miR-220-3p, F1, 31 = 3.48, P > 0.05; a significant miR-220-3p effect, P < 0.05; and a significant TMT effect, P < 0.001; for ROS: A significant interaction of TMT versus miR-220-3p, F1, 31 = 37.92, P < 0.001; a significant miR-220-3p effect, P < 0.01; and a significant TMT effect, P < 0.001; for protein carbonyl: A significant interaction of TMT versus miR-220-3p, F1, 31 = 11.07, P < 0.01; a significant miR-220-3p effect, P < 0.01; and a significant TMT effect, P < 0.001; for nitrite: A non-significant interaction of TMT versus miR-220-3p, F1, 31 = 37.92, P > 0.05; a non-significant miR-220-3p effect, P > 0.05; and a significant TMT effect, P < 0.001; for CAT: A significant interaction of TMT versus miR-220-3p, F1, 31 = 14.25, P < 0.001; a non-significant miR-220-3p effect, P > 0.05; and a significant TMT effect, P < 0.001; and for SOD: A significant interaction of TMT versus miR-220-3p, F1, 31 = 13.62, P < 0.001; a significant miR-220-3p effect, P < 0.05; and a significant TMT effect, P < 0.001).

Further assessment showed that TMT-induced hippocampal damage was associated with elevated levels of MDA [F (4, 30) = 15.53] (P < 0.001; an increase of 195.20%) (Figure 2A), intracellular ROS levels [F (4, 30) = 49.92] (P < 0.001; an increase of 545.60%) (Figure 2B), protein carbonyl [F (4, 30) = 12.68] (P < 0.01; an increase of 162.5%) (Figure 2C), and nitrite levels [F (4, 30) = 12.34] (P < 0.01; an increase of 146.84%) (Figure 2D) in the hippocampal region. Upon miR-220-3p treatment, significantly reduced levels of MDA (P < 0.05; a decrease of 35.49%), ROS (P < 0.001; a decrease of 61.97%), and protein carbonyl (P < 0.01; a decrease of 53.96%) were detected in the brain lysates versus the TMT-only cohort, but no significant effect was observed for nitrite levels (P > 0.05).

Moreover, TMT administration markedly decreased the activities of CAT (P < 0.001; a decrease of 62.12%) [F (4, 30) = 13.44] (Figure 2E) and SOD [F (4, 30) = 11.52] (P < 0.001; a decrease of 45.77%) (Figure 2F) relative to the control. Treatment of TMT-induced animals with miR-220-3p significantly increased the activities of CAT (P < 0.05; an increase of 86.84%) and SOD (P < 0.01; an increase of 61.74%) relative to the TMT-only cohort. In the meantime, the TMT group microinjected with NC mimic did not show a significant reduction in oxidative stress markers compared to the TMT cohort (P > 0.05). In addition, the miR-220-3p-treated control did not show a significant change in these oxidative factors, as compared to the control cohort (P > 0.05).

### 4.3. Inflammatory Markers

**Figure 3.**
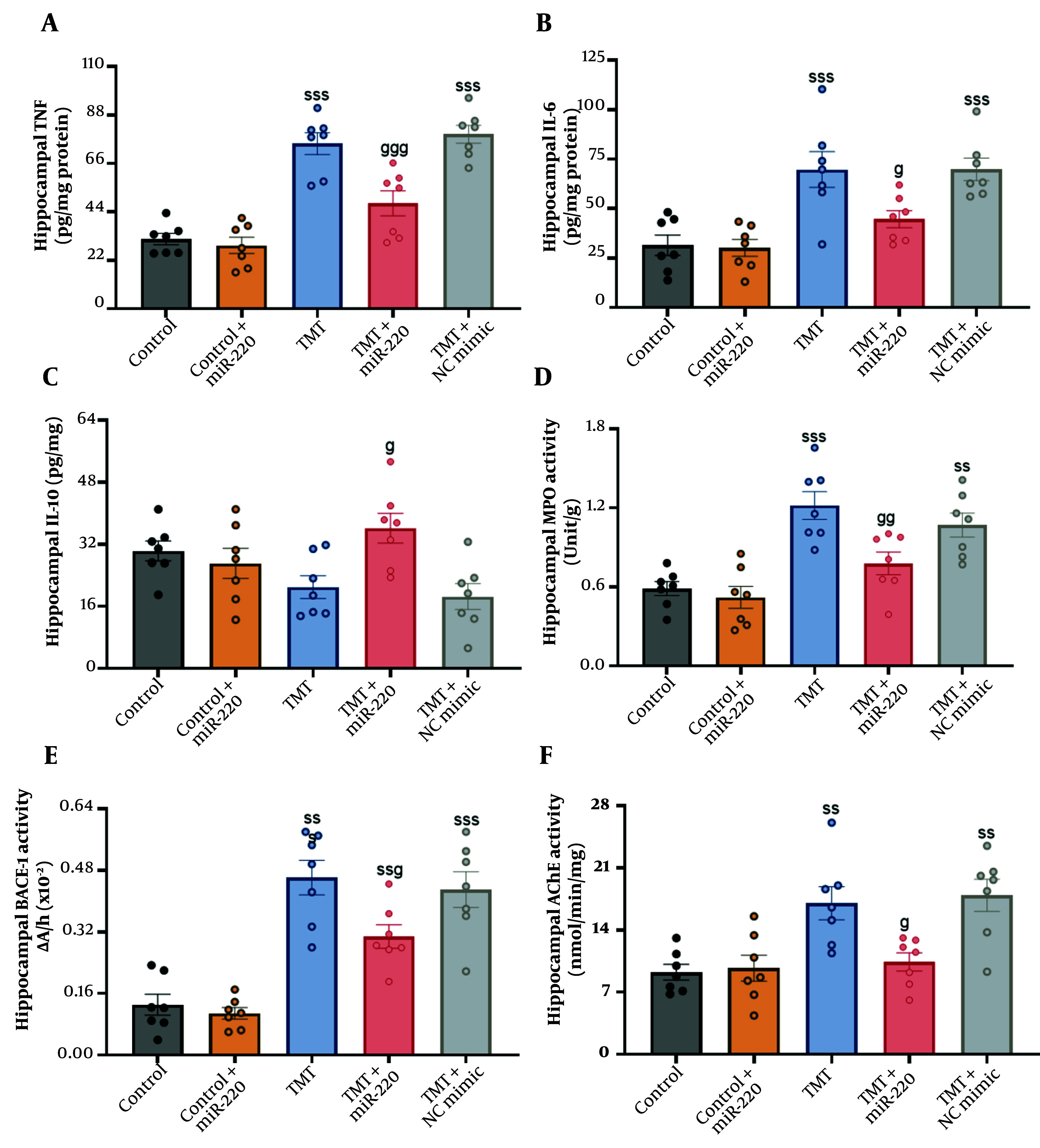
Impact of miRNA-220 on inflammatory markers in the hippocampal region of trimethyltin (TMT)-induced animals: A, tumor necrosis factor-α (TNF-α); B, interleukin-6 (IL-6); C, IL-10; D, myeloperoxidase (MPO); E, beta-secretase 1 (BACE-1); and F, acetylcholinesterase (AChE) (ss P < 0.01 and sss P < 0.001 vs. control; g P < 0.01, gg P < 0.01, ggg P < 0.001 vs. TMT).

Statistical analysis showed significant inter-group differences using two-way ANOVA (Figure 3 for TNF-α: A significant interaction of TMT versus miR-220-3p, F1, 31 = 10.92, P < 0.01; a significant miR-220-3p effect, P < 0.001; and a significant TMT effect, P < 0.001; for IL-6: A significant interaction of TMT versus miR-220-3p, F1, 31 = 4.69, P < 0.05; a significant miR-220-3p effect, P < 0.05; and a significant TMT effect, P < 0.001; for IL-10: A significant interaction of TMT versus miR-220-3p, F1, 31 = 9.94, P < 0.01; a significant miR-220-3p effect, P < 0.05; and a non-significant TMT effect, P > 0.05). Additional assessment showed that TMT induced an increase in the concentrations of TNF-α [F (4, 30) = 30.23] (P < 0.001; an increase of 137.18%) (Figure 3A) and IL-6 [F (4, 30) = 10.85] (P < 0.001; an increase of 121.67%) (Figure 3B), and a reduction in the concentrations of IL-10 [F (4, 30) = 4.49; a decrease of 30.63%] (Figure 3C) versus the control. In the TMT group treated with NC mimic, we also found increased levels of TNF-α (P < 0.001) and IL-6 (P < 0.001), as well as reduced levels of IL-10. Treatment with miR-220-3p suppressed the expression of TNF-α (P < 0.001; a decrease of 36.26%) and IL-6 (P < 0.05; a decrease of 35.99%) and improved the concentrations of IL-10 (P < 0.05; an increase of 72.15%) relative to the TMT-only cohort.

### 4.4. Activities of Myeloperoxidase, BACE 1, and Acetylcholinesterase

Statistical analysis showed significant inter-group differences using two-way ANOVA (Figure 3 for MPO: No significant interaction of TMT versus miR-220-3p, F1, 31 = 3.40, P > 0.05; a significant miR-220-3p effect, P < 0.05; and a significant TMT effect, P < 0.001; for BACE-1: No significant interaction of TMT versus miR-220-3p, F1, 31 = 3.22, P > 0.05; a significant miR-220-3p effect, P < 0.05; and a significant TMT effect, P < 0.001; for AChE: A significant interaction of TMT versus miR-220-3p, F1, 31 = 7.71, P < 0.01; a significant miR-220-3p effect, P < 0.05; and a significant TMT effect, P < 0.001).

Further assessment revealed significantly higher levels of MPO [F (4, 30) = 12.44] (P < 0.001; an increase of 108.62%) (Figure 3D), BACE-1 [F (4, 30) = 22.31] (P < 0.001; an increase of 253.84%) (Figure 3E), and AChE [F (4, 30) = 8.342] (P < 0.01; an increase of 84.18%) (Figure 3F) in TMT-induced animals compared with the controls. In the TMT group treated with the NC mimic, levels of MPO, BACE-1, and AChE were also increased (P < 0.01, P < 0.001, and P < 0.01, respectively). Moreover, significantly decreased levels of MPO (P < 0.01; a decrease of 36.36%), BACE-1 (P < 0.05; a decrease of 34.78%), and AChE (P < 0.05; a decrease of 38.90%) were observed in the miR-220-treated TMT-induced group in comparison with the TMT-only cohort.

### 4.5. Apoptotic and Pyroptotic Factors

**Figure 4.**
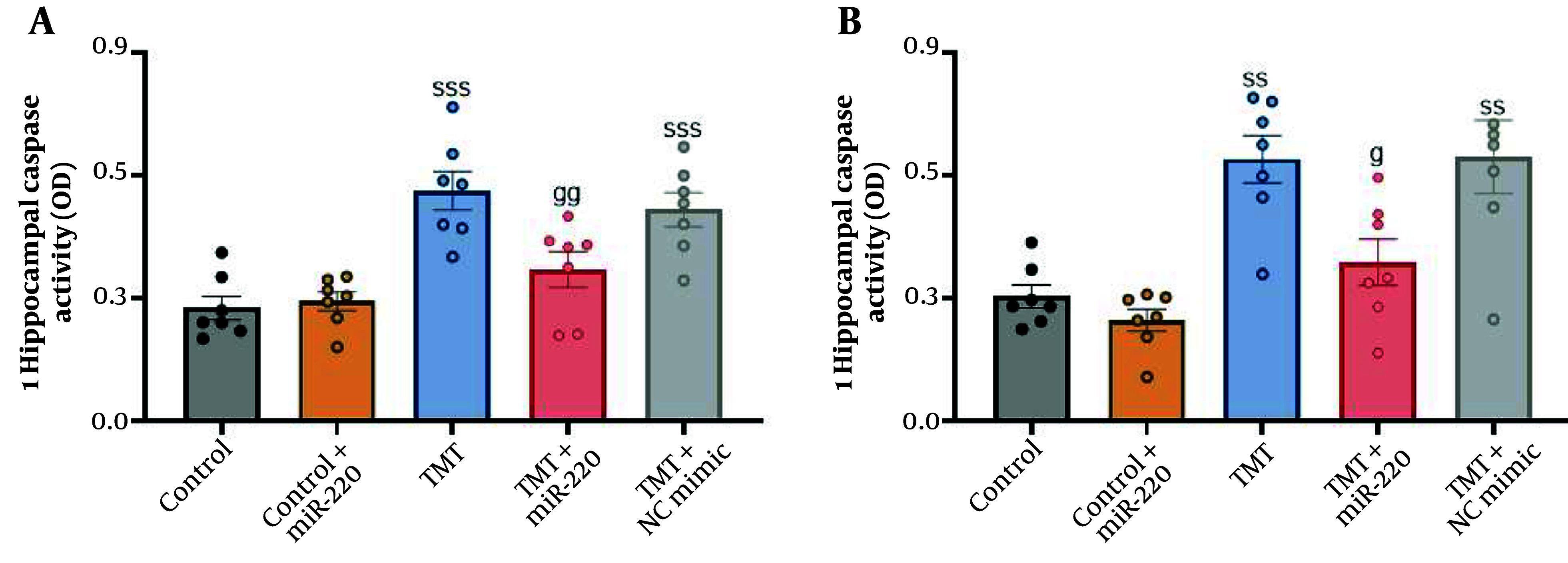
Impact of miRNA-220 on apoptotic factors in the hippocampal region of trimethyltin (TMT)-induced animals: A, caspase-1 and B, caspase-3 (ss P < 0.01 and sss P < 0.001 vs. control; g P < 0.01 and gg P < 0.01 vs. TMT).

To evaluate biomarkers of apoptosis and pyroptosis, we measured caspase-3 and caspase-1, respectively. Statistical analysis showed significant inter-group differences using two-way ANOVA (Figure 4 for caspase-1: A significant interaction of TMT versus miR-220-3p, F1, 31 = 6.97, P < 0.05; a significant miR-220-3p effect, P < 0.05; and a significant TMT effect, P < 0.001; for caspase-3: No significant interaction of TMT versus miR-220-3p, F1, 31 = 3.58, P > 0.05; a significant miR-220-3p effect, P < 0.01; and a significant TMT effect, P < 0.001). Additional assessment demonstrated that the activities of caspase-1 [F (4, 30) = 11.80] (P < 0.001; an increase of 157.40%) (Figure 4A) and caspase-3 [F (4, 30) = 11.03] (P < 0.01; an increase of 110%) (Figure 4B) were markedly increased in the TMT-only cohort compared with the control. In the TMT group treated with NC mimic, caspase-3 and caspase-1 levels were also elevated (P < 0.01 and P < 0.001, respectively). In addition, we found significantly reduced activities of caspase-1 (P < 0.01; a decrease of 33.92%) and caspase-3 (P < 0.05; a decrease of 74.60%) in TMT-induced animals treated with miR-220-3p compared with the TMT-only cohort.

### 4.6. Hippocampal Levels of Brain-Derived Neurotrophic Factor and Sirtuin-1

**Figure 5.**
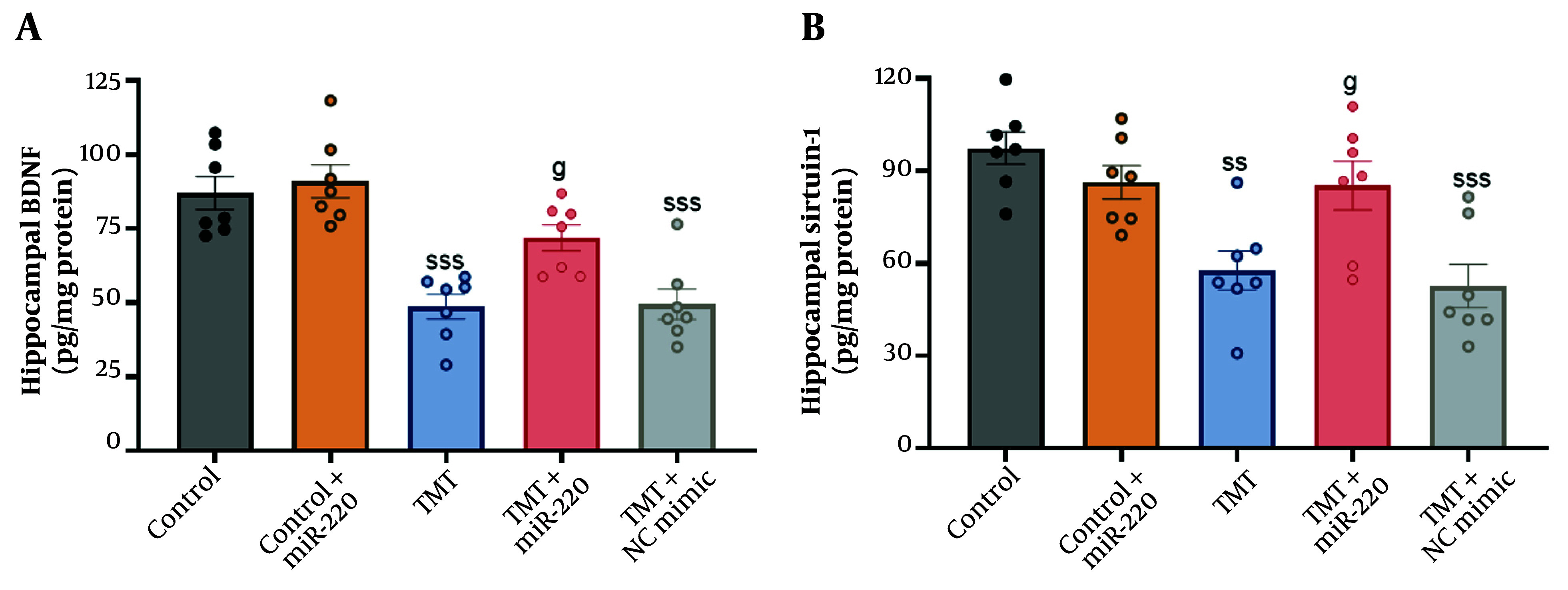
Impact of miRNA-220 on hippocampal, A, brain-derived neurotrophic factor (BDNF) and B, Sirtuin-1 in trimethyltin (TMT)-induced animals (ss P < 0.01 and sss P < 0.001 vs. control; g P < 0.05 vs. TMT).

Statistical analysis showed significant inter-group differences using two-way ANOVA (Figure 5 for BDNF: A significant interaction of TMT versus miR-220-3p, F1, 31 = 7.18, P < 0.01; a significant miR-220-3p effect, P < 0.05; and a significant TMT effect, P < 0.001; for Sirtuin-1: A significant interaction of TMT versus miR-220-3p, F1, 31 = 6.95, P < 0.05; a significant miR-220-3p effect, P < 0.05; and a significant TMT effect, P < 0.001). Additional assessment demonstrated that BDNF (P < 0.001) and Sirtuin-1 (P < 0.01) levels were significantly lower in the TMT group compared with the control. In contrast, miR-220 injection was associated with significantly higher levels of BDNF (P < 0.05) and Sirtuin-1 (P < 0.05) in the TMT group. In the TMT group treated with NC mimic, no significant change was observed for these factors.

### 4.7. Nissl Staining

**Figure 6.**
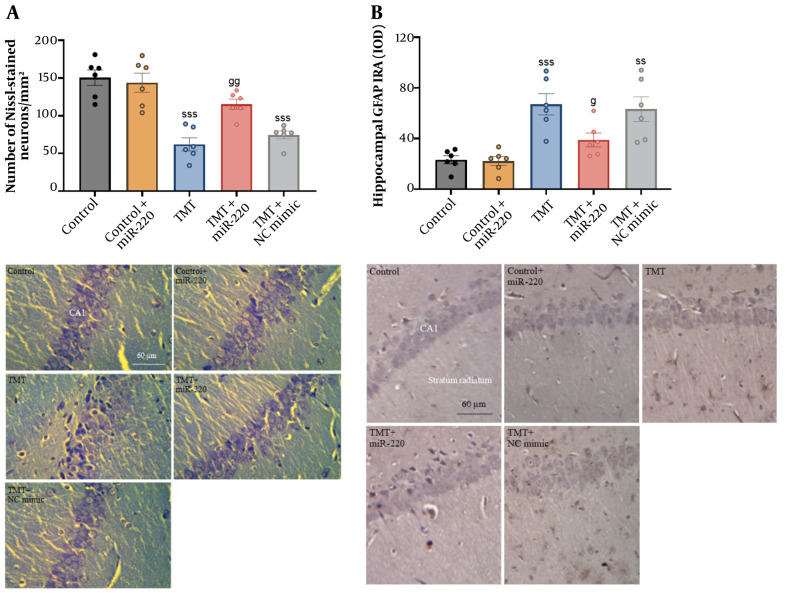
Impact of miRNA-220 on the loss of A, CA1 pyramidal neurons; and B, glial fibrillary acidic protein (GFAP) reactivity in trimethyltin (TMT)-induced animals (ss P < 0.01 and sss P < 0.001 vs. control; g P < 0.05 and gg P < 0.01 vs. TMT).

Statistical analysis showed significant inter-group differences using two-way ANOVA (Figure 6 a significant interaction of TMT versus miR-220-3p, F1, 26 = 9.98, P < 0.01; a significant miR-220-3p effect, P < 0.05; and a significant TMT effect, P < 0.001). Additional assessment demonstrated that TMT-induced animals exhibited neuronal damage and a reduced number of pyramidal neurons in the CA1 region (a decrease of 58.75%) [F (4, 25) = 19.24; P < 0.001] (Figure 6A). In the TMT group treated with NC mimic, the number of pyramidal neurons was also decreased (P < 0.001). Treatment with miR-220 prevented injury compared with the TMT-only cohort (an increase of 86.16%, P < 0.01).

### 4.8. Glial Fibrillary Acidic Protein Immunohistochemistry

Our statistical assessment showed significant inter-group differences using two-way ANOVA (Figure 6 a significant interaction of TMT versus miR-220-3p, F1, 26 = 4.33, P < 0.05; a significant miR-220-3p effect, P < 0.05; and a significant TMT effect, P < 0.001). Additional assessment showed greater GFAP reactivity in TMT-induced animals compared with the controls [F (4, 25) = 10.50] (P < 0.001; an increase of 189.09%). In the TMT group treated with NC mimic, GFAP reactivity was also increased compared with the controls (P < 0.01). Conversely, lower GFAP immunoreactivity was found in TMT-induced animals treated with miR-220-3p in comparison with the TMT-only cohort (a decrease of 60.97%, P < 0.05).

### 4.9. The miRNA-220-3p Gene Expression

Table 1 shows the relative gene expression of miR-220-3p in the hippocampal region one day after ICV miR-220-3p microinjection. Our unpaired *t*-test showed higher gene expression of miR-220-3p in the miR-220-3p-injected control group compared with the control (P < 0.05). This finding clearly indicates its hippocampal entry following ICV injection.

**Table 1.** Relative Gene Expression of miRNA-220-3p (N = 5 per Group)

Variable	Control	Control + miR-220
**Gene expression of miR-220-3p**	1 ± 0.16	1.63 ± 0.18 ^a^

Abbreviation: miR-220-3p, miRNA-220-3p.

^a^ P < 0.05 (versus the control group).

## 5. Discussion

The present investigation examined the therapeutic impact of miR-220-3p mimic on TMT-induced neurotoxicity and TMT-induced behavioral changes in rats. Our results revealed that TMT induction caused behavioral abnormalities, including impairments in spatial short-term and recognition memory. In addition, our findings demonstrated that TMT induction caused neuronal damage and a decreased number of pyramidal neurons in the CA1 region by escalating oxidative stress (increased levels of MDA, ROS, protein carbonyl, and nitrite, as well as reduced levels of CAT and SOD), elevating inflammation (increased concentrations of TNF-α and IL-6 and reduced concentrations of IL-10), and increasing the activities of MPO, BACE-1, AChE, and GFAP.

In this study, we assessed two possible target genes of miR-220-3p using bioinformatic tools, namely BDNF and Sirtuin-1. TMT is a hippocampal neurotoxin whose injurious effects are largely dependent on BDNF as an important factor in neuronal survival and synaptic plasticity. Previous research indicates that TMT exposure is associated with lower expression of BDNF, which is linked to cognitive decline (28, 29). Similarly, we also observed reduced hippocampal BDNF levels. In contrast, miR-220-3p microinjection was associated with the upregulation of BDNF. However, there is no report on this important issue, which requires further investigation.

Furthermore, from a mechanistic viewpoint, we also measured hippocampal Sirtuin-1 as another possible target of miR-220-3p. Our findings showed a lower level of Sirtuin-1 in the TMT-injured group. Consistent with this finding, TMT-induced neurotoxicity in the hippocampus is closely associated with the disruption of stress response cascades such as Sirtuin-1. This cascade plays important roles in the regulation of neuronal survival, inflammation, mitochondrial function, oxidative stress, and even synaptic plasticity (30, 31). Again, there is still no evidence on the direct effect of miR-220-3p on the expression of Sirtuin-1, which warrants additional investigation.

The TMT, a by-product of organotin compounds, is a neurotoxin commonly used to create in vivo models of neurodegenerative disorders such as AD, as it induces deficits in spatial recognition memory, increases neuronal excitability, and provokes seizure-like behaviors associated with derangements in the function of the hippocampal region of the brain (3, 6, 7). Owing to the selective neuronal damage relevant to behavioral changes, TMT is considered a suitable candidate for the establishment of cognitive–motor interference neurodegenerative disorders in animals (32). Previous investigations have indicated that spatial learning and memory deficits following TMT injection are linked to the susceptibility of the hippocampal region to TMT (33).

Our findings are consistent with previous investigations, as we also found that TMT injection caused behavioral abnormalities, such as disturbances in spatial short-term and recognition memory, which were improved by miR-220-3p. Behavioral abnormalities following TMT injection are associated with an elevation in oxidative stress. Kaur and Nehru found that the glutathione system in TMT-treated animals failed to adapt in response to oxidative stress, leading to an imbalance in redox status (9). The excessive generation of free radicals and ROS in TMT-treated animals causes damage to proteins, DNA, and lipids, thereby increasing levels of MDA as a byproduct of lipid peroxidation, as well as protein carbonyl and nitrite, and decreasing the activities of antioxidant enzymes such as CAT and SOD. This imbalance in redox status leads to increased activation of caspase proteins at both gene and protein levels and consequently increases neuronal death (4, 9, 34, 35). Liu et al. found that the detrimental impact of TMT in the hippocampus of mice is associated with the activation of caspase-dependent apoptosis and increased levels of cleaved caspase-3 (36). Yoneyama et al. also reported that caspase inhibitors may be considered effective therapeutic agents to attenuate TMT-induced cell death (37).

In this study, our data also demonstrate that TMT caused oxidative stress by increasing MDA, protein carbonyl, and nitrite levels and reducing the activities of antioxidant enzymes, and it escalated caspase-dependent apoptosis by elevating caspase-1 and caspase-3; all of these aberrations were reversed by miR-220-3p. In addition to oxidative stress, TMT-induced neurodegeneration and subsequent impairments in learning and memory are linked to suppressed hippocampal neurogenesis and increased neuroinflammation, mediated by the activation of microglial and astroglial cells and elevated levels of PCs and GFAP expression in the hippocampal region (11, 28, 38-40).

The TMT has demonstrated the capability to induce astrocyte polarization toward a pro-inflammatory phenotype, which in turn increases the level of neurotoxic complement C3 (C3) and the release of inflammatory mediators (TNF-α, IL-1β, and IL-6) in cell culture and animal models (41, 42). Moreover, TMT is capable of increasing the activity of MPO, a potent inflammatory enzyme biomarker released by neutrophils, which acts as a master regulator of neutrophils and subsequently promotes the release of inflammatory mediators in response to oxidative stress (43). Elevated oxidative stress also contributes to the production of BACE1, a key enzyme involved in generating Aβ oligomers and causing a detrimental cycle of inflammation and oxidative damage (44).

Our investigation also reveals that TMT could elevate neuroinflammation by increasing the concentrations of inflammatory mediators, GFAP expression, and the activities of MPO and BACE1, all of which were inhibited by treatment with miR-220-3p. In agreement with our findings, previous research has indicated that miR-220-3p is one of the key miRNAs involved in adaptation to hypoxia and plays a potent role in the synthesis of capillary-like structures from venous endothelial cells (20).

In line with our data, several investigations have found that miRNAs may serve as neuroprotective agents that mitigate neurodegeneration in neurological disorders such as AD by enhancing mitochondrial biogenesis (45), upregulating BDNF (46), inhibiting oxidative stress (47), and suppressing the pro-inflammatory response (48). Neurodegenerative diseases are characterized by the progressive loss of neuronal cells, with ensuing motor and cognitive complications. Abnormal expression of miRNAs has been reported in neurodegenerative diseases, indicating a pivotal role of miRNAs in these pathologies, including AD (49, 50).

Previous studies have shown altered expression of miRNAs in AD brain tissue in humans and animal models. Overall, many miRNAs may be involved in the pathogenesis of AD. Among these, some miRNAs are upregulated, whereas others are downregulated in brain tissue. Of relevance to this study, miR-298 and miR-328 can play significant roles by decreasing the expression of BACE1, an important contributor to Aβ overproduction in neuronal cells (51). In addition, increased expression of some miRNAs, such as miR-106a or miR-520c, can mitigate APP levels and BACE1 expression (52, 53). These results reveal that dysregulated miRNAs are associated with molecular pathways involved in AD pathogenesis, including impaired neurogenesis, insulin resistance, oxidative stress, and altered innate immunity. However, further studies are warranted to better demonstrate the therapeutic potential of miRNAs in AD pathophysiology.

The mechanistic insights underlying the effects of miR-220-3p in TMT-induced neurotoxicity likely involve multi-target regulation of oxidative and inflammatory signaling cascades. miR-220 may modulate redox homeostasis by targeting genes involved in ROS production and antioxidant defense, thereby restoring the balance between oxidants and scavenging enzymes such as SOD and CAT. Additionally, miR-220-3p may exert anti-inflammatory effects through the inhibition of transcription factors such as NF-κB and downstream cytokine networks, leading to decreased production of TNF-α, IL-6, and MPO activity. Given the observed suppression of BACE1 and GFAP expression, miR-220-3p might also interfere with amyloidogenic processing and astrocyte activation, further contributing to neuronal survival. These findings suggest that miR-220-3p acts through the coordinated regulation of oxidative stress, neuroinflammation, and apoptosis-related pathways to preserve hippocampal structure and function following TMT exposure.

Although the present study provides novel insights into the promising beneficial potential of miR-220-3p against TMT-induced hippocampal injury, several limitations should be acknowledged. First, the lack of experiments on direct target validation for the used miR mimic, including in silico target prediction tools, the dual-luciferase reporter assay as the gold standard method, and mRNA expression analysis of possible target genes using the RT-qPCR method, should be addressed in future studies in this field. However, protein-level validation was performed using ELISA assays for some target genes such as BDNF and Sirtuin-1. Second, only a single concentration and time point of both TMT and miR-220-3p were tested. Evaluating dose–response relationships and the long-term neuroprotective efficacy of miR-220-3p would provide a more comprehensive understanding of its therapeutic potential.

Third, although several standard behavioral tests (such as NOD and the Barnes maze) were used, additional paradigms assessing other cognitive domains (e.g., anxiety-like behavior, working memory, and locomotor activity) could strengthen the behavioral interpretation. Fourth, the study was conducted only on male Wistar rats. Considering potential sex differences in miRNA expression and neurotoxicity responses, the inclusion of both sexes and possibly different animal models would improve generalizability. Fifth, the ICV delivery of the miR-220-3p mimic ensures precise targeting but is invasive and not directly applicable to clinical use. The development of less invasive delivery systems, such as nanoparticle- or exosome-mediated transport across the blood–brain barrier, should be explored.

Future studies should aim to further elucidate the direct molecular targets and signaling pathways regulated by miR-220 to confirm its mechanistic role in neuroprotection. Employing transcriptomic or proteomic profiling could help identify downstream effectors involved in oxidative stress and inflammatory regulation. Moreover, investigations using human neuronal or induced pluripotent stem cell (iPSC)-derived models would be valuable to verify translational relevance. Exploring alternative, less invasive delivery methods, such as exosome- or nanoparticle-based systems, could also enhance therapeutic feasibility. Finally, long-term behavioral and histopathological studies are needed to determine whether miR-220-3p confers sustained functional recovery, which may position it as a promising candidate for intervention in neurodegenerative disorders such as AD.

### 5.1. Conclusions

In this investigation, miR-220-3p demonstrated notable benefits in mitigating TMT-induced neurotoxicity by ameliorating spatial learning and memory impairments. Here, miR-220-3p exerted its beneficial effects through the inhibition of TMT-induced neuronal damage by suppressing oxidative stress (reducing ROS levels and lipid peroxidation while improving the activities of antioxidant enzymes), neuroinflammation (decreasing the release of inflammatory mediators, GFAP expression, and the activities of MPO and BACE1), and caspase-dependent apoptosis and pyroptosis in the hippocampal region.

## Data Availability

The datasets generated and analyzed in the present study will be available from the corresponding author upon reasonable request.
